# Assessment of the static upright balance index and brain blood oxygen levels as parameters to evaluate pilot workload

**DOI:** 10.1371/journal.pone.0214277

**Published:** 2019-03-28

**Authors:** Jicheng Sun, Shan Cheng, Jin Ma, Kaiwen Xiong, Miao Su, Wendong Hu

**Affiliations:** Deparment of Aerospace Medicine, The Fourth Military Medical University, Xi’an, China; University of Canberra, AUSTRALIA

## Abstract

**Objective:**

To investigate the potential for static upright balance function and brain-blood oxygen parameters to evaluate pilot workload.

**Methods:**

Phase 1: The NASA Task Load Index (NASA-TLX) was used to compare the workloads of real flights with flight simulator simulated flight tasks in 15 pilots (Cohort 1). Phase 2: To determine the effects of workload, 50 cadets were divided equally into simulated flight task load (experimental) and control groups (Cohort 2). The experimental group underwent 2 h of simulated flight tasks, while the control group rested for 2 h. Their static upright balance function was evaluated using balance index-1 (BI-1), before and after the tasks, with balance system posturography equipment and cerebral blood oxygen parameters monitored with near infrared spectroscopy (NIRS) in real time. Sternberg dual-task and reaction time tests were performed in the experimental and control groups before and after the simulated flight tasks.

**Results:**

(Phase1) There was a significant correlation between the workload caused by real flight and simulated flight tasks (*P*<0.01), indicating that NASA-TLX scales were also a tool for measuring workloads of the stimulated flight tasks. (Phase 2) For the simulated flight task experiments, the NASA-TLX total scores were significantly different between the two groups (*P*<0.001) and (pre-to-post) changes of the BI-1 index were greater in the experimental group than in controls (*P*<0.001). The cerebral blood oxygen saturation levels (rsO_2_) (*P*<0.01) and ΔHb reductions (*P*<0.05) were significantly higher in the experimental, compared to the control group, during the simulated flight task. In contrast to the control group the error rates (*P* = 0.002) and accuracy (*P*<0.001) changed significantly in the experimental group after the simulated flight tasks.

**Conclusions:**

The simulated flight task model could simulate the real flight task load and static balance and NIRS were useful for evaluating pilots’ workload/fatigue.

## Introduction

Pilot fatigue, which can have physical or mental causes, is considered an internal risk factor for unsafe acts, because it negatively affects the human operator's internal state [1, 2]. A leading cause of pilot fatigue is task workloads caused by sustained cognitive work [3–5]. This study focuses on pilot workload using a continuous task load model and also evaluates the potential of static balance and NIRS to evaluate workload and, potentially, fatigue.

Physical fatigue is the transient inability of a muscle to maintain optimal physical performance, the main symptoms of which include lack of power and dexterity [6]. Mental fatigue is a transient decrease in maximal cognitive performance resulting from prolonged periods of cognitive activity [3–5, 7].

It can manifest as somnolence, lethargy or loss of directed attention [8–10]. Physical or mental load can contribute to pilot fatigue, which may result in a slower response, inattention and even mistakes that can lead to accidents [11]. The assessment of fatigue can be divided into subjective and objective methods [12]. Some subjective methods use tools to judge the presence and degree of workloads, including the NASA Task Load Index (NASA-TLX) scale [13], the Subjective Workload Assessment Technique (SWAT) scale [14], and the Cooper-Harper questionnaire [15]. Objective methods determine fatigue mainly by measuring changes in body functions, which comprise measurements of physiological, psychological and biochemical indicators, and working performance tests. For example, ophthalmotropometry and electroencephalography (EEG) are used for physiological indicators, reaction time test and critical flicker-fusion (CFF) test for psychological indicators, measurements of corticosteroid levels under workload by radioimmunoassay (RIA) for biochemical indicators, and the Sternberg dual-task test for working performance tests. Subjective methods are applied widely and have good reliability and validity, but the results can be affected by the individual’s motivations and experiences, and they do not necessarily accurately reflect pilot fatigue [16]. Some studies also indicate that participants cannot accurately estimate their ability to perform their duties, which may result in overconfident and inaccurate judgment [17].

Electroencephalography (EEG) and heart rate variability (HRV) can be used as objective methods to judge pilot fatigue in the laboratory, since EEG α,β, β/α and (α+θ)/β index changes have been detected in fatigued subjects while performing a simulated driving task [18], and HRV monitoring in a flight crew revealed that a higher workload score was associated with high frequency component reductions [19]. However, their evaluation is complex and difficult to interpret, and it would be impractical to apply these methods in the field [20]. Many biochemical indicators have no unequivocal meaning in the assessment of central fatigue, and it is possible to obtain contradictory results from the same biochemical index [21]. Therefore, finding accurate, noninvasive and convenient methods to assess pilot fatigue and establish warning systems to prevent accidents would be highly desirable. Ergonomic studies on postural control, subjective workload assessment, and psychomotor performance have been used for assessing fatigue caused by sleep deprivation. These methods include, amongst other things, the measurement of changes in the upright balance function [22] and cerebral blood oxygen saturation before and after sleep deprivation [20]. We explored whether these methods could be used to assess pilot workload, which may lead to fatigue.

The static upright balance function test was originally developed to assess patients with vestibular dysfunction, and this method has been used in rehabilitation medicine to evaluate the status of patients with brain disease, and the effects of physical and cognitive rehabilitation training in these patients [23, 24]. However, research has shown that some indexes of the upright balance function change under a task load [25].

Near-infrared blood oxygen spectroscopy is a novel method for monitoring blood oxygen parameters in specific tissues. This method is non-invasive and convenient, and it is currently applied in neonatal, intensive care and sports medicine [26–28]. Some studies have described the application of near-infrared techniques to measure blood oxygen parameters to detect muscle fatigue [29], while others have focused on the role of changing cerebral blood oxygen parameters for the detection of driver fatigue [30]. Based on these studies and on the advantages of the near-infrared technique, we analyzed the changes occurring in pilots’ cerebral blood oxygen parameters and applied this technique to establish a novel method to detect and assess pilot workload and possible task induced fatigue.

After we established a simulated flight task load model, we compared the subjective feelings of workload caused by this model with that caused by real flight missions through the NASA-TLX scale, to verify the effectiveness of the simulated model. Next, we tested the pilots’ static upright balance function before and after the task load, while at the same time monitoring cerebral blood oxygen parameters during the task load. We then verified our hypothesis by confirming that these two methods could effectively detect pilot workload.

## Materials and methods

The protocols of this study were approved by the Research Ethics Committee of Fourth Military Medical University. Written, informed consent was obtained from all of the participants prior to their participation in our study.

### Phase 1

Fifteen pilots (**Cohort 1)** of fighter planes, who met the flight permission criteria, were recruited and participated in real aircraft flight based on visual flight rules during daytime, which were of medium difficulty level and these 15 pilots also completed the simulated flight tasks. NASA-TLX scales were evaluated for these 15 pilots after their real-flight and simulation flight tasks to confirm the consistency of flight workload between the tasks and confirm that the established simulated flight tasks model could truly reflect the workload caused by the flight missions.

### Phase 2

**Fig 1:**
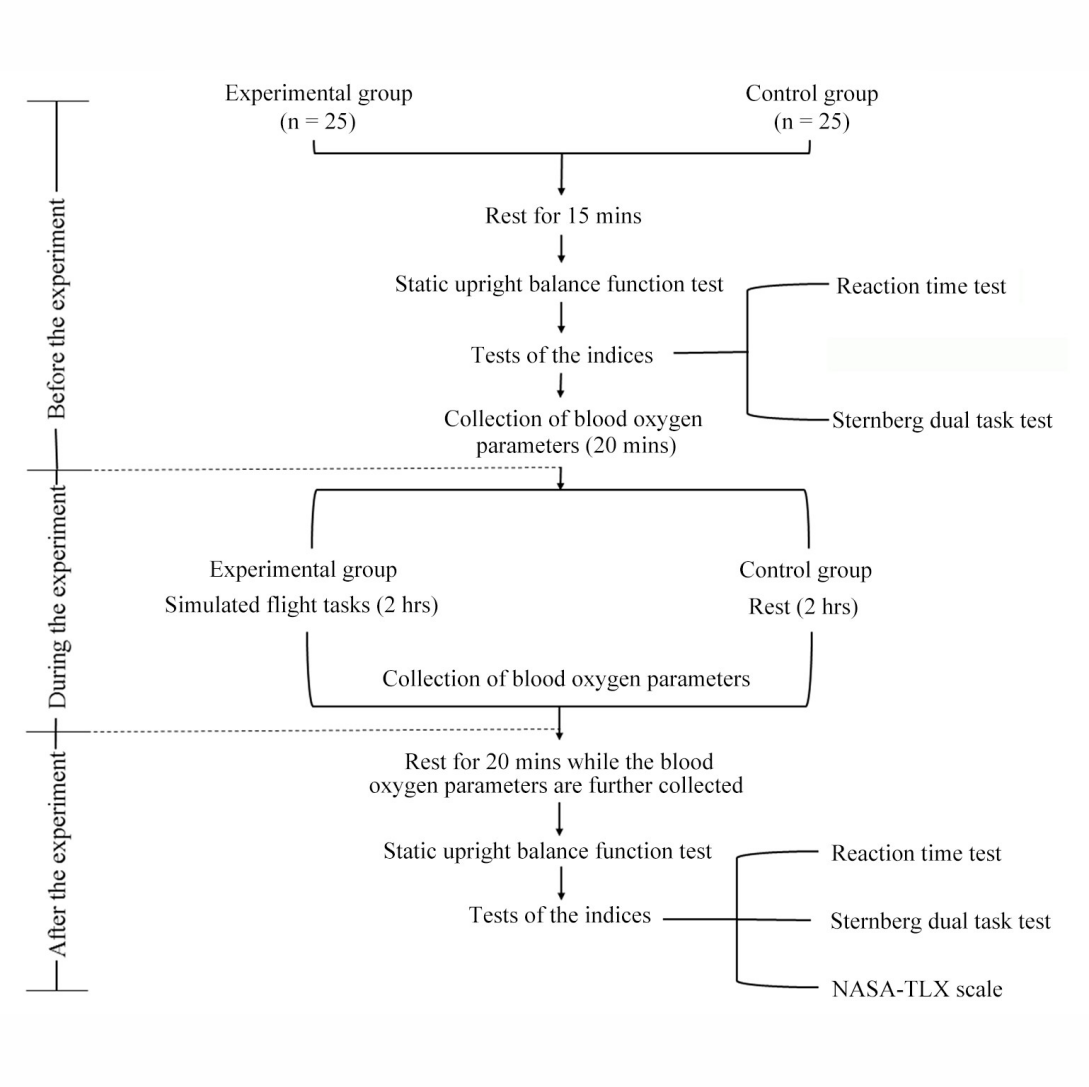
Flow chart of the experimental procedures.

Fifty male military cadets (**Cohort 2**) were recruited and randomly allocated into a control group (n = 25) and a flight simulation group (experimental group, n = 25). They were not allowed to consume any drugs or drinks that would affect the central nervous system, such as coffee or strong tea, for two days before the tasks. Both groups underwent related workload assessments before and after the task, or the rest period accordingly. In the previous study, 2 h of task load was needed to affect the participants’ physiological and psychological functions [31]; therefore, we set the duration of the tasks to 2 h, between 18:00 and 22:00 every day, with two participants (one in the control group and one in the experimental group). The participants must not have performed any intense activities like running, playing basketball or football and other intense physical activities for two days before the experiment, and they had 1 h to learn and practice the simulated flight task load one day before the experiment, in order to minimize the learning effect on task performance. On the day of the assessment, two participants were tested in two different rooms. The protocol of the experiment is shown in Fig 1.

### Simulated flight tasks (flight simulator)

The simulated flight tasks were performed in a flight simulator. These man-machine multitasks required the participants to complete four different tasks, including monitoring and controlling the instrument persistently, performing emergency tasks, continuously tracking the flight target, and performing other tasks as required. The participants used a joystick and keys with their hands to complete the tasks, and the first three tasks were treated preferentially, with the remaining capacity to be used for any other tasks.

### Methods to measure pilot workload

#### NASA-TLX scale

The NASA-TLX scale is a multi-dimensional table for task load assessments developed by NASA [32]. It has six factors, which are mental demand, physical demand, temporal demand, performance, effort, and frustration. The participants completed a self-assessment on each factor, with a greater score indicating a greater load. The participants also ranked the degree of correlation between workload and the factor, giving different weights to these six factors. The weights were 1/21, 2/21, 3/21, 4/21, 5/21, and 6/21, respectively. The total score represented the task load index, with a greater total score indicating a higher total load level (S1 Fig).

#### Sternberg dual-task test

This test includes two tasks, which are a short-term memory task and a cursor-tracking task. The left hand is used to respond to the short-term memory task by pressing a button, while the right hand is used for the cursor-tracking task by controlling the joystick. The performance of both hands was recorded and analyzed by the computer program Matlab 2010a (Mathworks, Natick, MA, USA). The duration of this test was adjustable and was set to 2 min after 2 min practice in the present study. All participants were right-handed.

#### Reaction time test

This test was performed using a system that consisted of two parts: a display interface and a transponder. The display interface contained nine round LED lamps (three red, three yellow and three green). The transponder contained three buttons that represented each color. One LED lamp was turned on pseudo randomly by the Java SE 7.0 (Sun Microsystems, Santa Clara, CA, USA) software, and the participants had to press the corresponding button. If the participant pressed the right color, then the lamp was turned off and another one was turned on; however, if the participant pressed the wrong button, the lamp would not be turned off until the correct one had been pressed. The duration of this test was set to 2 min, and the accuracy as well as the error rate was recorded.

### Testing the static upright balance function

In a previous study, we modified an instrument that can evaluate the static upright balance function of participants under different conditions (TETRAX of Israel Sunlight Medical Ltd., Israel) by developing a new software based on their hardware and software [33] with indicators that target to sleep status, etc. The modified software calculation method mainly relies on principal component analysis and we found that there was a specific posture that brought the most dramatic change in the parameters of the static upright balance function during the task load, which followed a linear trend. Based on the findings, we extracted the most sensitive parameters by principal component analysis and obtained a comprehensive balance index-1 (BI-1) to measure the changes occurring in balance functions during a work task. For vision, vestibular sensation or lower proprioception measurements of the participants, the tester could obtain and analyze different parameters from different standing postures.

In the present study, the participants were barefoot and after opening their eyes they looked straight ahead on a crosshair on the wall which was adjusted for height and the testing duration was 32 s. We calculate the static balance function index by using the parameters sensitive to the task load under the standing on the mat with eyes open position; these parameters included eight frequency domains and 13 time domain parameters for a total of 21 parameters. The frequency domain parameter divided the body's swaying frequency into eight frequencies (F1 to F8): 0.01–0.1 Hz, 0.1–0.25 Hz, 0.25–0.35 Hz, 0.35–0.5 Hz, 0.5–0.75 Hz, 0.75–1 Hz, 1–3 Hz, 3 Hz, and > 3 Hz. The 13 time domain parameters included: the gravity distribution parameters of the front and back of both feet (WD); namely, the gravity distribution (%) of the left heel, left sole, right heel, and right sole, recorded as WD1, WD2, WD3 and WD4 (%), circumference area (CA); rectangle area (RA); effective value area (EVA); whole path length (WPL); unit area path length (UAPL); left and right deviation distance (Mx); front and back deviation distance (My); standard deviation (SDx) in the left and right directions; standard deviation (SDy) in the front and back direction.

### Monitoring cerebral blood oxygen parameters

We used a near-infrared monitor for non-invasive blood oxygen parameter measurements, with a portable probe (the near-infrared tissue blood oxygen parameter non-destructive monitoring instrument TASH-100, developed by Tsinghua University), as the sensor, which could be directly attached to the surface of the tissue to measure blood oxygen parameters of it [34]. Every participant had their cerebral blood oxygen parameters recorded every 2 s for 160 min. The data were divided into eight time intervals (20 min each), in order to reveal the overall trend of any changes. The values of the blood oxygen parameters were averaged over 20 min and the average values in each time interval represented the data characteristics of that time interval. The first 20 min of data were regarded as the basal resting state, and every other time interval’s data were compared with the data in the first 20 min. The changes in cerebral blood oxygen parameters in the experimental group were then compared with the changes occurring in the control group.

The measured parameters were the weighted average of the blood oxygen parameters of the arterioles, venules and capillaries; that is, the blood oxygen parameters of the tissue. We measured the blood oxygen parameters of the forehead (at the position of EEG electrode Fp1), which represents the cerebral blood oxygen supply according to the literature [35]. The main parameters were: change in deoxyhemoglobin concentration (ΔHb); change in oxygenated hemoglobin (ΔHbO_2_); change in total hemoglobin concentration (ΔtHb, ΔtHb = ΔHb + ΔHbO_2_); and blood oxygen saturation (rsO_2_) of the brain tissue.

### Statistical analysis

The data were analyzed using SPSS ver. 16.0 (IBM, Armonk, NY, USA) and continuous variables are presented as mean ± SD. Differences between control and experimental groups and differences between before and after measurements were compared using variance analysis of repeated measurements. Because of the failure of some of the instruments during the experiments, some of the cadets’ data could not be included in the analysis. Thus, we analyzed the data from 21 participants in the control group and 24 participants in the experimental group. Categorical variables were summarized as a percentage of the whole and a chi-squared test was used to detect differences. A repeated measures analysis was used to compare the changes in the time-domain and frequency-domain indicators. The static upright balance function were used to construct a static upright balance index (SUBI) using a principle component analysis [33].

Results of the pilot's task load evaluation of the NASA scale for real and simulated flights were analyzed by Pearson correlation analysis. Curve estimation was used to analyze the relationship between indicators and pilot workload. In addition, ANOVA was used to analyze continuous variables between three or more groups and Student’s t test was used for analyzing continuous variables between two groups. *P* < 0.05 was considered to be statistically significant.

## Results

### Baseline characteristics of the participants

Table 1 shows the baseline characteristics of participants in the 2 cohorts of the study.

**Table 1:** Baseline information of the participants.

	Phase 1 Cohort 1	Phase 2 Cohort 2		
	Experimental group (n = 15)	Experimental group (n = 24)	Control group (n = 21)	*P*-value*
Age (years)	27.00 ± 1.23	21.10 ± 0.89	21.23 ± 0.74	0.600
Weight (kg)	67.34 ± 6.38	65.60 ± 8.44	66.20 ± 4.32	0.771
Height (m)	1.78 ± 4.56	1.76 ± 4.67	1.75 ± 5.31	0.995
BMI (kg/m^2^)	22.03 ± 1.89	21.62 ± 2.45	22.11 ± 1.76	0.451
Sleep time before the test (h)	7.33 ± 1.35	7.54 ± 1.65	7.88 ± 2.09	0.546

*Indicates P values of differences between experimental and control groups in cohort 2.

### Phase 1

#### Correlation between the simulated and the real flight tasks

The 15 enrolled pilots for the real flight task were male with an average real flight duration of 1.62 ± 0.51 h (Table 2). We found that there was a linear association between real flight task loads and simulated flight task loads in mental demand, effort, and total points for the NASA-TLX scale, which means that there is a positive correlation between the workload caused by the simulated flight tasks and the real flight tasks.

**Table 2:** NASA-TLX scale towards the real flight task load and the simulated flight task load (n = 15, $\bar{x}$ ± s).

	Real flight task load (n = 15)	Simulated flight task load (n = 15)	Correlation index (r)	*P*-value
Flight duration (h)	1.62 ± 0.51	1.50 ± 0.00	-	-
Mental demand	3.42 ± 0.96	3.71 ± 1.34	0.477	< 0.01
Physical demand	2.03 ± 1.35	0.90 ± 0.97	-0.207	> 0.05
Temporal demand	1.25 ± 0.93	2.24 ± 1.12	0.203	> 0.05
Performance	1.09 ± 0.62	1.22 ± 0.84	0.398	> 0.05
Effort	3.02 ± 1.16	3.03 ± 1.25	0.564	< 0.05
Frustration	0.71 ± 0.40	1.32 ± 0.70	0.434	> 0.05
Total points	11.52 ± 2.52	12.43 ± 2.22	0.773	< 0.01

### Phase 2

#### Effectiveness of multiple assessment methods of pilot workload using the simulated flight task load model

**NASA-TLX scale:** The NASA-TLX total scores in the experimental group was higher than those in control group after the task (*P* < 0.001) (Table 3).

**Table 3:** The result of the NASA-TLX scale, Sternberg dual task and reaction time test before and after the task load in the experimental and control groups.

		Experimental group (n = 24)			Control group (n = 21)			*P*-value
		Before task	After task	After-before	Before task	After task	After-before	Experimental vs control
NASA-TLX total score		-	13.73 ± 2.27	-	-	10.64 ± 2.14	-	< 0.001
Sternberg dual task	Response performance	5.01 ± 1.55	5.02 ± 2.28	0.02 ± 1.6	5.31 ± 2.26	6.04 ± 2.53	0.72 ± 1.48	0.142
	Track performance	5.07 ± 1.93	5.45 ± 1.83	0.36 ± 1.82	5.75 ± 2.13	5.72 ± 1.42	-0.03 ± 1.78	0.462
Reaction time test	Accuracy	64.31 ± 6.48	60.58 ± 5.81*	-3.73 ± 3.25	63.90 ± 3.74	64.42 ± 4.21	0.52 ± 2.71	< 0.001
	Error rate	2.15 ± 1.36	3.19 ± 1.96	1.04 ± 1.49	2.41 ± 2.08	1.81 ± 1.05	-0.60 ± 1.71	0.002

*P < 0.05, when after the task compared to before the task in the same population.

**Sternberg dual-task test:** Comparing pre-to-post test changes of the response and tracking performances, there were no significant differences in the answer and trace scores (*P* = 0.142 and *P* = 0.462, respectively) between the experimental and control group.

**Reaction time test:** The differences in performance on the reaction time tests before and after the tasks, including the rates of correct and erroneous responses, were greater in the experimental than in the control group (*P* < 0.001 and *P* = 0.002). The rate of correct responses in the experimental group dropped significantly and the error rate increased. In the control group, however, the rate of correct responses did not change significantly (Table 3).

These results indicate that the NASA-TLX scale and the reaction time test can be used as assessment tools for the simulated flight task load model and may reflect the workload state of participants.

#### The static balance function test was able to detect workload caused by the simulated flight tasks

For the eight frequency and 13 times domain parameters of PO, significantly statistical differences in 2 h were revealed by the following seven parameters: F1, F3, F4, F6, WD2, EVA and SDy, which could be used in the calculation of balance index 1 (BI-1) that can reflect the task load level under the standing on the mat with eyes open position [31]. BI-1 = 3.065 × F1 + 5.346 × F3 + 13.161 × F4 + 21.954 × F6 + 37.446 × WD2 + 115.454 × EVA + 114.183 × SDy + 23.746. (Note: The index in the formula is the original parameter of the participants under the standing on the mat with eyes open position)

BI-1 values were not significantly different before the simulated flight task in the experimental and control groups. The pre-to-post BI-1 change was significantly higher in the experimental group (44.71 ± 2.93 to 48.10 ± 3.72, *P* < 0.01), whereas in the control group it did not change significantly (44.17 ± 3.61 to 43.86 ± 3.31), leading to a significant difference of BI-1 value changes between the experimental and control groups (3.39 ± 3.65 vs -0.31 ± 1.46, *P* < 0.001) after the simulated flight task load.

#### Correlations between changes in cerebral blood oxygen parameters and the workload state, as well as physiological and psychological changes

**Fig 2:**
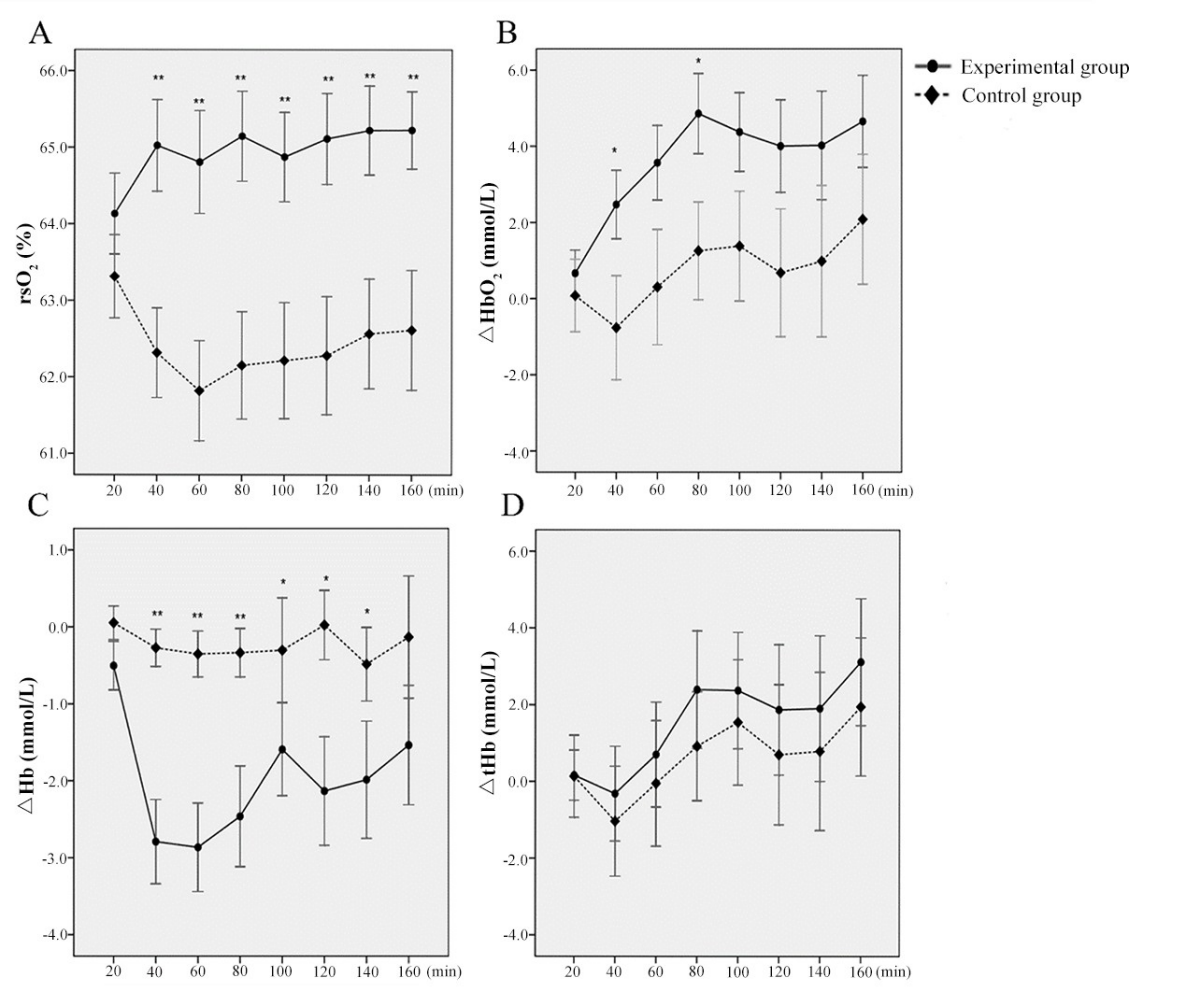
The cerebral blood oxygen parameters in experimental and control groups every 20 min within 160 min. (A) blood oxygen saturation (rsO_2_); (B) Changes of HbO_2_ (ΔHbO_2_); (C) changes of deoxyhemoglobin concentration (ΔHb); (D) changes in total hemoglobin concentrations (ΔtHb).

There was no statistical difference in cerebral blood oxygen parameters between the two groups when they were in the resting state (rsO_2_, *P* = 0.287; ΔHbO_2_, *P* = 0.598; ΔHb, *P* = 0.165; ΔtHb, *P* = 0.983). However, the data in the following 140 min showed that the cerebral blood oxygen saturation (rsO_2_) of the experimental group was significantly higher than that of the control group (F_40_ = 10.35, *P* < 0.01; F_60_ = 10.02, *P* < 0.01; F_80_ = 10.87, *P* < 0.01; F_100_ = 7.93, *P* < 0.01; F_120_ = 8.69, *P* < 0.01; F_140_ = 8.45, *P* < 0.01; F_160_ = 8.25, *P* < 0.01) (Fig 2A). For Δ HbO_2_, there was no significant difference between the two groups in the first 20 min, but ΔHbO_2_ was significantly higher at 40 min and 80 min in the experimental than in the control group (F_40_ = 4.09; *P* < 0.05; F_80_ = 4.79, *P* < 0.05 (Fig 2B). Similarly, ΔHb in the control group was not significantly different with the experimental group during the first 20 min, but the △Hb reduction was significantly higher (△Hb was lower) in the experimental than the control group in the following 120 min (F_40_ = 16.04, *P* < 0.01; F_60_ = 13.78, *P* < 0.01; F_80_ = 7.82, *P* < 0.01; F_100_ = 6.27, *P* < 0.05; F_120_ = 6.23, *P* < 0.05; F_140_ = 4.86, *P* < 0.05; Fig 2C). However, there were no significant differences in ΔtHb between the two groups in the following 160 min (Fig 2D). Therefore, we found that rsO_2_, ΔHbO_2_, and ΔHb are effective parameters for evaluating workload.

**Fig 3:**
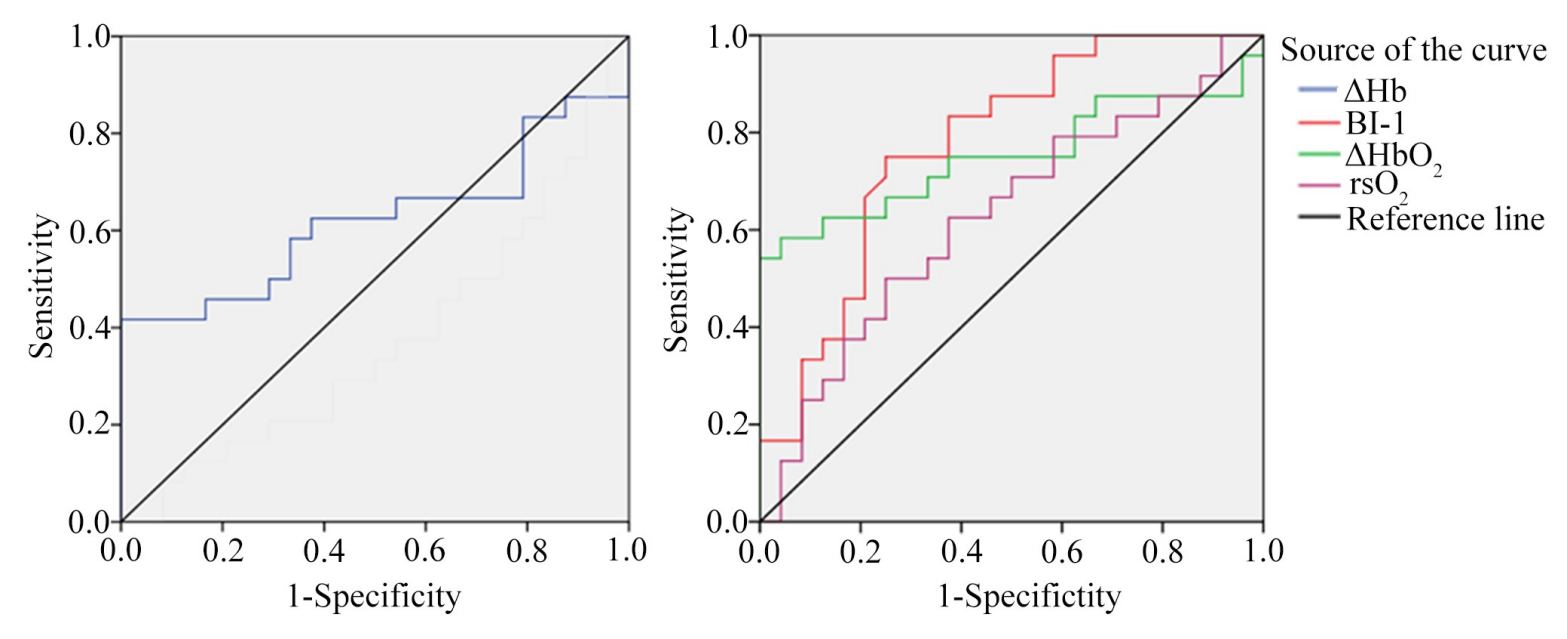
The ROC curve of 4 different indicators for measuring the task load.

Moreover, we used the data from BI-1, rsO_2_, ΔHbO_2,_ and ΔHb to obtain receiver operating characteristic (ROC) curves to evaluate those parameters’ effectiveness in assessing workload and analyzed the cut-off value of each index. We then analyzed the accuracy and specificity of each index to determine the practical significance of each index and to select the one that could be used to assess the degree of workload. We found the ΔHbO_2_ and ΔHb values had the highest specificities (95.80%), and BI-1 had the highest value for accuracy (Fig 3 and Table 4).

**Table 4:** The cut-off value of each index.

Indicators	Cut-off value	Accuracy (%)	Specificity (%)
Cerebral blood oxygen saturation (rsO_2_)	0.648	62.50	62.50
Change in oxyhemoglobin (ΔHbO_2_)	4.510	58.30	95.80
Change in deoxyhemoglobin (ΔHb)	-2.380	41.70	95.80
Balance index-1 (BI-1)	46.040	75.00	75.00

## Discussion

These results indicate that there was higher cerebral blood oxygen saturation in the experimental group during Phase 2, which means that rsO_2_ can be used as an index to assess the workload of a pilot. In addition, we found that ΔHbO_2_ and ΔHb could reveal the physical condition of a pilot. Thus, using a near-infrared monitor for recording non-invasive blood oxygen parameters may be used as a novel method to assess pilot workload.

Based on a simulated flight task load model that could simulate a real flight task, this study used two novel workload assessment methods to assess the physiological and psychological state of pilots: (1) measuring their static upright balance function and (2) monitoring their cerebral blood oxygen parameters, while these parameters might also reflect pilot fatigue. Flying a plane is a demanding profession, and it is very important to develop a convenient and accurate assessment and warning system to avoid accidents caused by pilot fatigue.

Based on our previous study, we hypothesized that the BI-1 portion of the BI could be applied to assess the workload caused by flight tasks in pilots [31]. Currently the static upright balance function test is mostly used to measure athlete’s fatigue. For example, Armstrong and Yaggie (2004) studied the relationship between the balance index and lower limb fatigue. They found that lower limb fatigue negatively affected the BI of the athletes, who needed some time to recover [36]. Likewise, the pilots participating in our experiment felt tired, both psychologically and physically; therefore, the BI-1 index changed significantly after the task, compared to before the task. This result was consistent with the results of previous studies related to changes in brain cognition which affected the postural stability [33, 37, 38]. In addition, in the present study we applied principal component analysis to obtain BI-1. This method could standardize the original multidimensional parameters of the static upright balance function test, and identify the most sensitive parameters. This means that greater weight can be given to those parameters in which more significant changes occur in response to fatigue [39, 40]. Although previous studies have demonstrated that principal component analysis can improve the validity of related predictions [41, 42], we still need to investigate further the distribution pattern of BI-1 in more pilots and modify the index, creating a relevant reference standard to make it possible to establish a fatigue warning system and a standard to evaluate both the physiological and psychological state of pilots before flight. In addition, for the Sternberg dual-task test, though in this study only right handed participants were included, for further studies with new participants prior reliability data related to dominant handedness of this particular test must be derived.

The oxygen saturation of specific tissues can reflect the dynamic balance between oxygen consumption and supply in the microcirculation, and the cerebral oxygen saturation level can reflect the real oxygen metabolism of brain tissue [43, 44]. Our results indicated a significant difference in changes in cerebral oxygen saturation between the experimental and control groups. Prefrontal cortex oxygen saturation rose most significantly during the first 20 min of a task load, when compared to the base value before the task. Furthermore, ΔHb had the most significant change in the first 20 min of the simulated flight tasks indicating that the brain tissue needed to increase oxygen consumption quickly, by raising the level of oxygen metabolism, to ensure that the tasks could be accomplished. These changes may be related to some compensatory mechanism produced by the body.

However, in the 80–100 min (time point 5), the cerebral oxygen saturation declined to some degree and the value of ΔHbO_2_ also decreased, while the value of ΔHb increased somewhat compared to the previous time point. We also found that the cerebral oxygen saturation rose slowly after 80 min. Some experiments indicated that there is a correlation between the occurrence of fatigue and the decline of oxygen saturation in certain tissues [45–47]. Once the amount of blood oxygen is not able to meet the needs of the brain, cerebral hypoxia occurs. Short-term hypoxia can cause fatigue, lethargy, nausea and other symptoms. Under normal conditions, there is some physiological fluctuation of the oxygen saturation in brain tissue, but the amplitude of the fluctuation is not large. The symptoms of fatigue in participants might have resulted from short-time brain hypoxia caused by the flight tasks, as changes in the blood oxygen saturation of brain tissue in the experimental group was basically consistent with this phenomenon. The results of the present study also show that our method can be used to monitor blood oxygen fluctuations in the brains of pilots. It could also be used to analyze the physiological and psychological status of the pilots, and to identify the time point at which they start to feel increased workload.

Limitations of the present study were the limited number of participants and the duration of the task load was only 2 h. In addition, although we used the NASA-TLX scale to compare the workload caused by simulated and real flight tasks, the simulated and real flight tasks still have some differences that may cause different psychological effects in the pilots like motivation, emotion, degree of stress and arousal [48].

## Conclusion

This study discussed some novel ideas and suggested new possibilities for developing such a system, providing relevant evidence for the assessment of pilots’ physiological and psychological states. We believe that the model of the human-machine multitasks, which simulated the real flight tasks used in this study, accurately reflected the workload state of the pilots when they were performing real flight tasks. We identified some changes in the subjective scales and operational performance of the pilots detected by error rate and accuracy tests, and we believe that these changes confirm that the model we built is able to simulate pilot workload at some level. The feasibility and accuracy of BI-1 in addition to cerebral blood oxygen parameters and reaction time tests confirmed our hypothesis that this index can be used to assess pilots’ increased workload. Nevertheless, additional studies should be performed in the future with a longer duration of simulated flight tasks and a larger number of pilots. Such studies will make it possible to use different groups with different task lengths to confirm the feasibility of BI-1, as well as changes in cerebral blood oxygen parameters in assessing pilot workload.

## Supporting information

S1 Fig Contents of NASA-TLX scale. (TIF)
