# Supplementary figures and images for: Assessment of the static upright balance index and brain blood oxygen levels as parameters to evaluate pilot workload
Source: PLoS One. 2019 Mar 28;14(3):e0214277. doi: 10.1371/journal.pone.0214277 (PMC6438667; doi:10.1371/journal.pone.0214277)

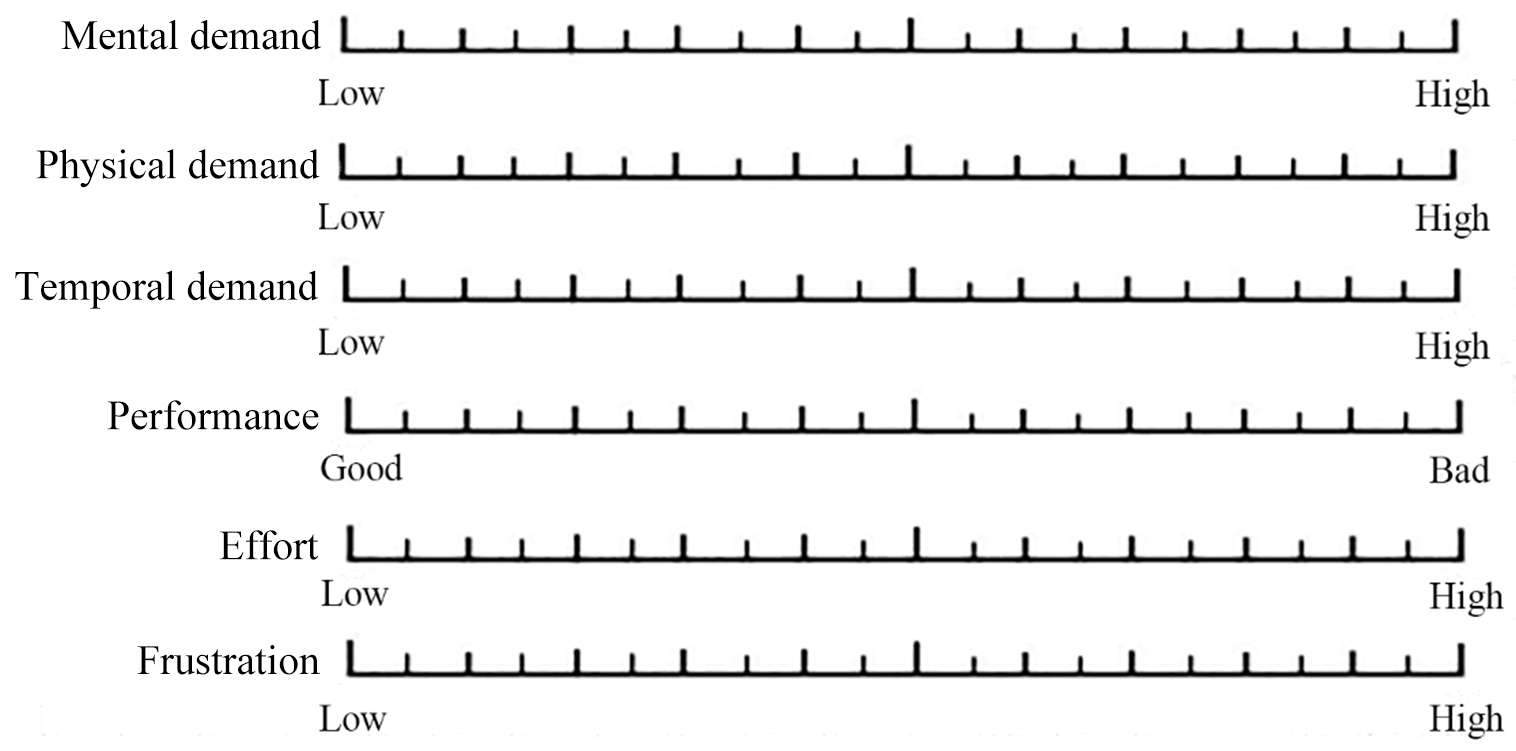

Supplement: S1 Fig — (TIF) [file pone.0214277.s001.tif]
